# Electrochemical Study of Polymer and Ceramic-Based Nanocomposite Coatings for Corrosion Protection of Cast Iron Pipeline

**DOI:** 10.3390/ma11030332

**Published:** 2018-02-25

**Authors:** Ameen Uddin Ammar, Muhammad Shahid, Muhammad Khitab Ahmed, Munawar Khan, Amir Khalid, Zulfiqar Ahmad Khan

**Affiliations:** 1 School of Chemical & Materials Engineering, National University of Sciences & Technology, Islamabad 46000, Pakistan; ameenuddin03@gmail.com (A.U.A.); mshahid@scme.nust.edu.pk (M.S.); khitab.770@gmail.com (M.K.A.); engr.munawarkhan@gmail.com (M.K.); amir_ms08@scme.nust.edu.pk (A.K.); 2 Bournemouth University, NanoCorr, Energy & Modelling (NCEM) Research Group, Poole House P123, Talbot Campus, Fern Barrow, Poole BH12 5BB, UK

**Keywords:** nanocomposite coating, corrosion, Electrochemical Impedance Spectroscopy (EIS), seawater, crude oil, few layers grapheme Polyvinyl alcohol (PVA), Titanium Oxide (TiO_2_), direct current (DC), alternating current (AC), Polyaniline (PANI)

## Abstract

Coating is one of the most effective measures to protect metallic materials from corrosion. Various types of coatings such as metallic, ceramic and polymer coatings have been investigated in a quest to find durable coatings to resist electrochemical decay of metals in industrial applications. Many polymeric composite coatings have proved to be resistant against aggressive environments. Two major applications of ferrous materials are in marine environments and in the oil and gas industry. Knowing the corroding behavior of ferrous-based materials during exposure to these aggressive applications, an effort has been made to protect the material by using polymeric and ceramic-based coatings reinforced with nano materials. Uncoated and coated cast iron pipeline material was investigated during corrosion resistance by employing EIS (electrochemical impedance spectroscopy) and electrochemical DC corrosion testing using the “three electrode system”. Cast iron pipeline samples were coated with Polyvinyl Alcohol/Polyaniline/FLG (Few Layers Graphene) and TiO_2_/GO (graphene oxide) nanocomposite by dip-coating. The EIS data indicated better capacitance and higher impedance values for coated samples compared with the bare metal, depicting enhanced corrosion resistance against seawater and “produce water” of a crude oil sample from a local oil rig; Tafel scans confirmed a significant decrease in corrosion rate of coated samples.

## 1. Introduction

Corrosion of various structural components and service pipelines are quite common in industrial applications. A huge amount of resource and effort is continually dedicated to increasing the service life of components since corrosion causes significant loss in terms of costs and most importantly in human life [1]. Many techniques are used for protecting the structures against corrosion: corrosion inhibitors [2,3]; cathodic and anodic protection [4,5]; design modification, alteration with environment and most importantly changing the material which is probably more corrosion resistant e.g., stainless steel [6]. Application of various types of protective coatings is another popular method for protecting metals from aggressive corrosion reactions [7,8]. Researchers are continuously seeking new innovative coatings which will enhance corrosion protection of assets in conventional environments [9].

Nanocomposite materials are high-performance materials having at least one of the phases consisting of dimension <100 nm [10]. In the current study, PVA/PANI/FLG, a polymeric nanocomposite coating and TiO_2_/GO, a ceramic nanocomposite coating, have been investigated for protection of cast iron pipeline material against corrosion in seawater and “produced water” from crude oil wells. Each constituent of the coatings is believed to contribute towards improvement in corrosion resistance, as previously discussed by Fontana et al. [11]. It has been demonstrated that vinyl polymers remain unaffected in low acidic and alkaline environments and it also reveals low water absorption behavior of PVA as it behaves as a corrosion inhibitor by forming complexes and covering surfaces to protect the metallic material from corrosion [12]. Corrosion protection has been attributed to increased corrosion potential [7]. The high electrical resistivity of TiO_2_ indicates its capability to provide reasonably good corrosion protection [13]. Additionally, graphene, which was used as reinforcement in both coatings, has also demonstrated its significant anticorrosion properties [14] owing to its hydrophobic nature due to its non-polar covalent double bond [15]. The current study discusses the relative corrosion resistance performances of the two coatings. 

Electrochemical Impedance Spectroscopy (EIS) is considered to be a powerful technique which uses a small perturbation AC signal to study an electrochemical cell [16]. EIS technique was used to characterize the interface of the metal immersed in electrolyte using a Potentiostat. Data obtained from EIS generated two plots: the first, a Bode plot in which magnitude of impedance and phase shift was plotted against frequency; the second, Nyquist plot where imaginary impedance was plotted against real impedance. Corrosion resistance of the coatings was analyzed using both plots.

## 2. Experimentation

### 2.1. Sample Preparation

An industrial pipeline cast iron sample was used for investigation; the samples were ground using 80, 120 and 240 grit papers in order to generate a relatively smooth surface of the samples [17].

### 2.2. Synthesis of Coating Material 

#### 2.2.1. PVA/PANI/FLG

Materials used in the synthesis of this coating are given in Table 1.

##### Preparation of Polyaniline

100 mL of deionized water was mixed with Aniline monomer and 1 mol HCl solution was slowly added. Ammonium peroxydisulfate was added as an initiator and appearance of green color indicated that the polymerization was completed. The solution was washed several times and then dried to get the required Polyaniline powder.

##### Preparation of PVA Solution

The 100 mL amount of deionized water was heated to 90 °C, followed by the addition of PVA powder while the solution was continuously stirred on a magnetic stirrer (D0310 Analog Magnetic Stirrer, Labnet Internatiional, Edison, NJ, USA) at a constant speed of 500 RPM. A clear solution of PVA was obtained after 20 min.

##### Preparation of Nanocomposite Solution

Deionized water was heated to 90 °C and PVA was slowly added until a complete clear solution was obtained; it was followed by slow addition of PANI till the color of the solution changed to dark green. In the next step the required amount of FLG was added in the solution when PVA and PANI was completely dissolved. The selected amount for PVA/PANI/FLG nanocomposite in percentages was 90/10/0.5 respectively. The solution was then kept on a sonication probe for 45 min to disperse the PANI. The complete process has been described elsewhere [18].

#### 2.2.2. TiO_2_/GO Coating

##### Synthesis of TiO_2_ Nanoparticle 

TiO_2_ nanoparticles were formed by the sol-gel method using titanium tetra isopropoxide (TTIP) as precursor; ethanolamine was used to enhance the stability (linker) of the sol, and methoxyethanol was used as solvent to exhibit good mechanical stability and electrochemical response. An inert environ ment was provided to an empty three-necked flask for 15 min. TTIP (≥97%) of Sigma Aldrich, Saint Louis, MO, USA, 2-methoxyethanol (99.9+ %), and ethanolamine (99+ %) in a fixed molar ratio (1:4:0.5) were introduced to the three-necked flask, refluxed and stirred at room temperature for 1 h under an inert atmosphere. Then it was heated at 80 °C for 1 h. Temperature of the solution was increased to 120 °C and the stirring was maintained for 2 h at the same temperature. This technique resulted in a clear solution indicating complete dissolution of the precursors in the solvent.

##### Synthesis of TiO_2_/Graphene Composite Solution

The solution of 0.5 mg/1 of graphene oxide in 2-methoxyethanol was prepared and sonicated for 50 min in an ultrasonic water bath in order to ensure good dispersion of the graphene oxide. This graphene oxide solution was mixed in the already-prepared TiO_2_ solution. The resulting mixture was heated in an electric oven at 60 °C for 20 h [19].

### 2.3. Dip Coating

The samples were coated with respective nanocomposite coating by the dip-coating technique; the samples were initially dipped in the coating solution and kept in the solution for about 2 min, however, the step was repeated to achieve desired thickness. After drainage of the surplus solution, the samples were kept in a furnace (below T_g_ of polymers) to evaporate the solvent and to obtain the deposited thin films. The parameters were set to acquire the required thickness of the coatings in accordance to the Landau Levich equation for dip-coating [20]; the thickness of the coatings were estimated to be about 5 µm.

### 2.4. Electrochemical Corrosion Studies 

The electrochemical study was performed by employing the Gamry^®^ framework. EIS studies were performed using a two-electrode cell and corrosion behavior diagrams were generated using the three-electrodes system which helped to determine corrosion rate of the coated and uncoated samples. The three-electrodes electrochemical measurement system has been described in various locations, e.g., [21,22], which follows the theory explained by Fontana and Greene [23]. The coated sample was made as one electrode; SCE (Saturated Calomel Electrode) was used as a reference, and a graphite rod was employed as a counter electrode. The Gamry^®^ framework, which encases all required apparatuses such as a potentiostat, a zero-resistance ammeter and a sweep generator, all controlled by its software module DC105™, has a built-in capability of not only generating “E-log I” diagrams but also calculating corrosion rate. EIS300™ of the Gamry^®^ framework was used for Electrochemical Impedance Spectroscopy of coated samples, employing a two-eletrode system where the counter electrodes also serve for the reference [24]. 

Two types of electrolytes were used for corrosion investigation: (1) seawater obtained from Karachi, Pakistan (pH 7.9 and conductivity 51–53 mS/cm); and (2) “produced water” obtained from a natural gas well in Khyber Pakhtoon Khah, Pakistan (pH 7.6 and conductivity 148–152 µS/cm). Sample exposed surface areas were around 3 × 3 cm^2^ and kept immersed for about 40 min before starting electrochemical testing. 

## 3. Results

### 3.1. Equivalent Circuit Modelling

**Figure 1:**
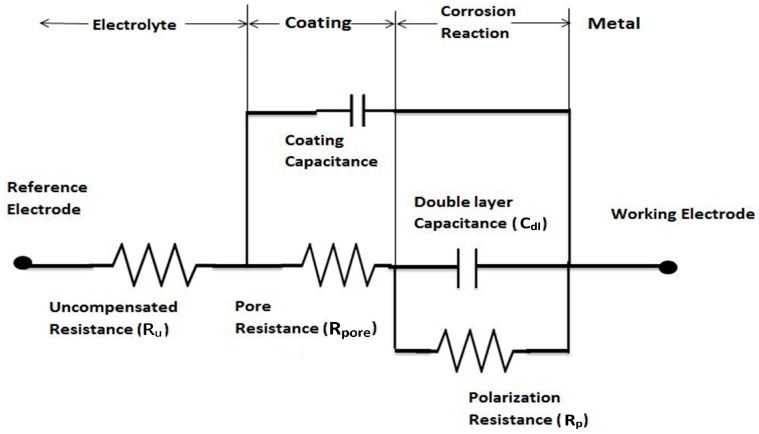
Equivalent circuit for a coated metal [16].

The equivalent circuit model for a coated metal as documented in the literature is given in Figure 1, which displays various portions of the electrochemical cell and their behaviors.

### 3.2. EIS in Seawater 

**Figure 2:**
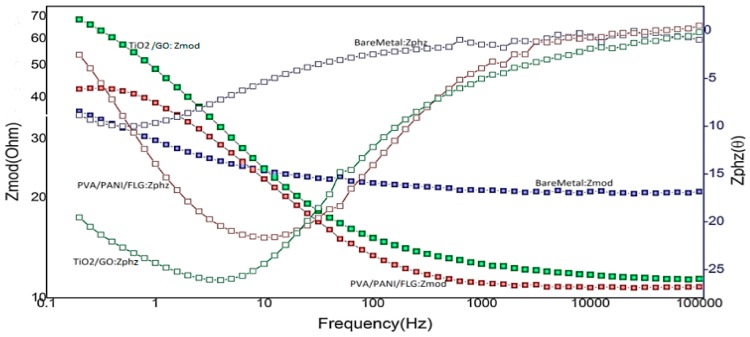
Comparison of Bode plots (in seawater).

Figure 2 displays Bode plots of uncoated and coated samples with PVA/PANI/FLG and TiO_2_/GO coatings. As revealed by the graph, both the coatings showed significant improvement in corrosion resistance. Between the two coatings, the TiO_2_/GO coating showed the higher resistance in seawater compared with PVA/PANI/FLG as displayed by higher value of impedance of this coating and a noticeable difference in phase shift. The data suggested that in seawater TiO_2_/GO nanocomposite coating was more resistant against corrosive ions and resisted ionic attack before degradation. However, PVA/PANI/FLG coating displayed relatively low resistance to corrosion and provided lower protection, as evident from its comparison with the other coatings and the bare metal; an early degradation of this coating was evident compared with the other coating.

**Figure 3:**
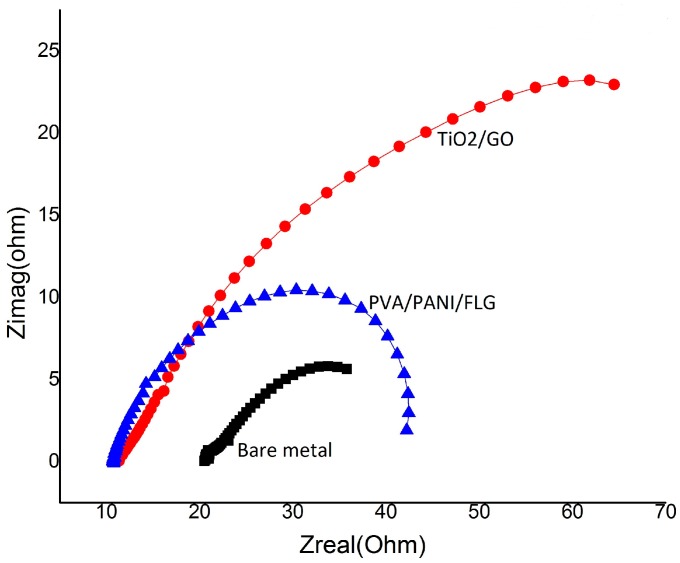
Comparison of Nyquist plots (in seawater).

Figure 3 shows Nyquist plots for the three cases. As evident from the figure, TiO_2_/GO demonstrated the highest resistance in terms of coating strength before it got degraded compared with PVA/PANI/FLG and the bare sample, which appeared inferior to the both coatings. Although PVA/PANI/FLG coating showed a relatively higher impedance indicating greater protection, however, the impedance values were far lower than the TiO_2_/GO coating. 

Table 2 shows that pore resistance (R_pore_) for TiO_2_/GO coating appeared to be higher compared with PVA/PANI/FLG coating, indicating better coating stability. It can be attributed to lower population of pores generated during exposure of metal to the electrolyte as the corrosion was suppressed of the polymeric coating; pores are considered to play a significant role in increasing or decreasing the corrosion reaction. Polymeric coating showed a higher coating capacitance (C_c_) compared with ceramic coating as indicated by lower impedance, since the two properties are inversely proportional. It was also believed that polymeric coating with higher capacitance can store higher charge, thereby degrading the coating faster as compared to the ceramic base coating exhibiting lower coating capacitance.

### 3.3. DC Corrosion Testing in Seawater 

**Figure 4:**
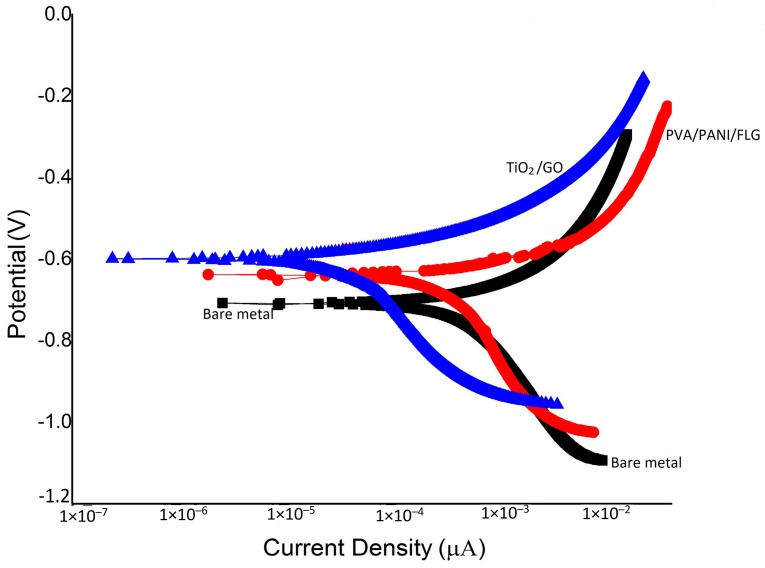
Tafel Scans in seawater: Current density in µA/cm^2^; Potential (V vs. SCE).

Figure 4 reveals “E-log I” curves for coated and uncoated metallic samples exposed to seawater, and the corrosion rates determined from these diagrams are given in Table 3.

**Figure 5:**
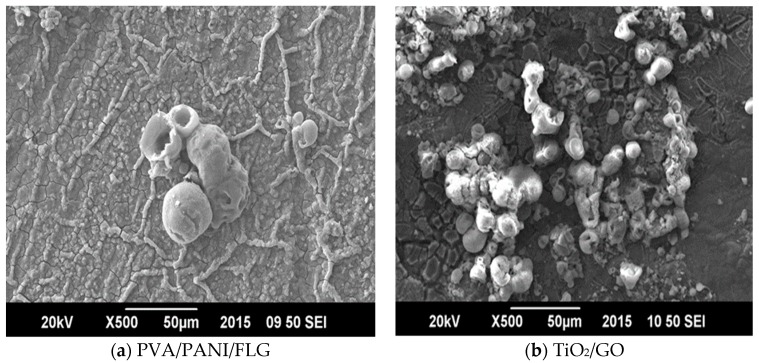
SEM micrographs of coated samples exposed to seawater. (**a**) PVA/PANI/FLG; (**b**) TiO_2_/GO.

The comparison of bare and coated metallic samples indicates that both the coatings provided a significant decrease in the corrosion rate of cast iron. Individually, the PVA/PANI/FLG coating reduced the corrosion rate to one half of that of the bare metal, whereas the TiO_2_/GO coating caused a significant decrease in the corrosion rate, amounting to 15 times less than the bare metal. Due to better stability, the corrosion rate of TiO_2_/GO coating in seawater happened to be seven times better than the PVA/PANI/FLG coating when exposed to seawater. It was believed that the seawater resulted in pitting, leading to the development of microcracks of the PVA/PANI/FLG polymer coating, presumably due to the presence of chloride ions [11]. The chloride ion, however, did not prove to be aggressive towards the ceramic base coating, revealing higher resistance. Figure 5 reveals the coatings after electrochemical tests in the seawater environment and it is evident from the SEM images that both the coatings were observed to be distorted and ruptured due to the vigorous attack of corrosive ions; cracks were evident, which widened the pores resulting in the sample becoming exposed and the corrosion reinitiated.

### 3.4. EIS in “Produced Water” from an Oil Exploration Plant

**Figure 6:**
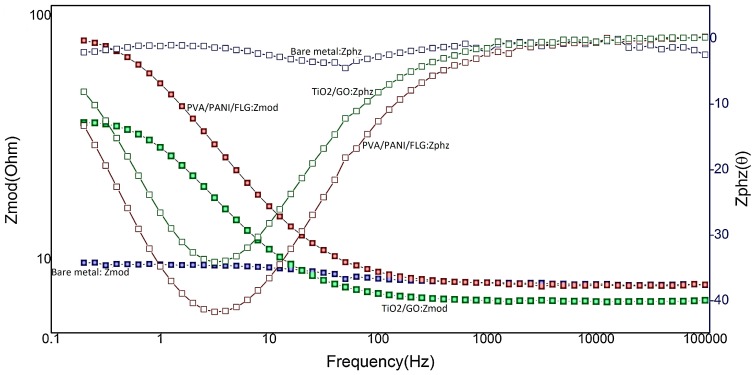
Comparison of Bode Plots (produced water).

Figure 6 displays a Bode plot of coated and uncoated samples exposed to “produced water”. The bare metal appeared to be significantly weak in magnitude and the phase shift as indicated by two horizontal lines. It was also evident from Figure 5 that PVA/PANI/FLG polymeric coatings appeared to be offering higher protection in “produced water” compared with ceramic-based TiO_2_/GO coatings, as evident form the value of impedance and the degree of phase shift for polymeric coating sample. The greater the stability of coating in the corrosive environment surely offered more corrosion resistance to the PVA/PANI/FLG coated sample then the ceramic coating. 

**Figure 7:**
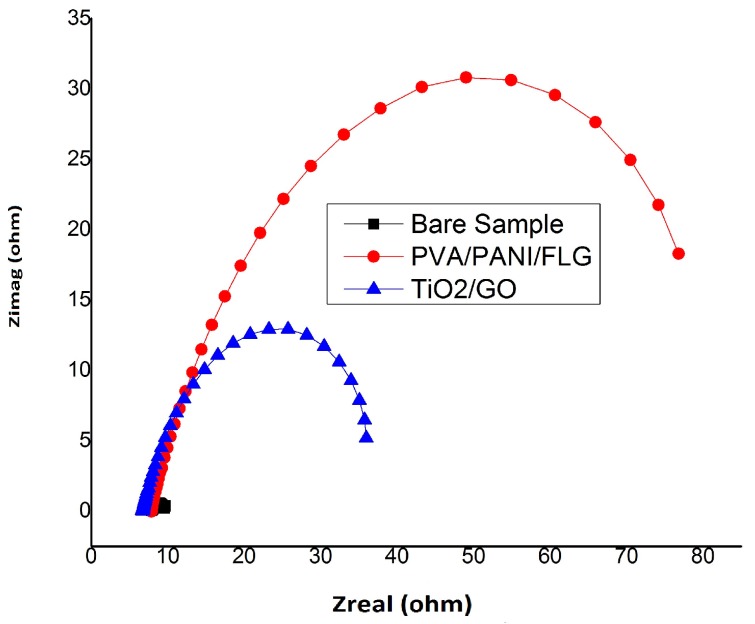
Comparison of Nyquist Plot (produced water).

The Nyquist plots for the three samples is shown in Figure 7, which also supports the findings of Figure 6, i.e., the PVA/PANI/FLG coated sample offers a higher stability in the corrosive environment of the crude oil, while TiO_2_/GO coating, although inferior compared with the polymeric coating, appears to afford significantly higher protection compared with the bare sample.

Pore resistance and coating capacitance value for PVA/PANI/FLG obtained was 303.9 × 10^−3^ ohms and 17.59 × 10^−3^ F respectively as shown in Table 4. While 166.1 × 10^−3^ ohms pore resistance and 28.79 × 10^−3^ F coating capacitance was observed for the case of TiO_2_/GO as mentioned in Table 4. The data reveals that relatively higher value of pore resistance for PVA/PANI/FLG indicates better stability when exposed to the “produced water” environment, offering higher impedance thereby hindering pores’ generation [9]. On the other hand, coating capacitance of the ceramic-based coating appeared to be higher, indicating a lower value of impedance resulting in lower resistance against pores’ generation.

### 3.5. DC Corrosion Testing in “Produced Water”

**Figure 8:**
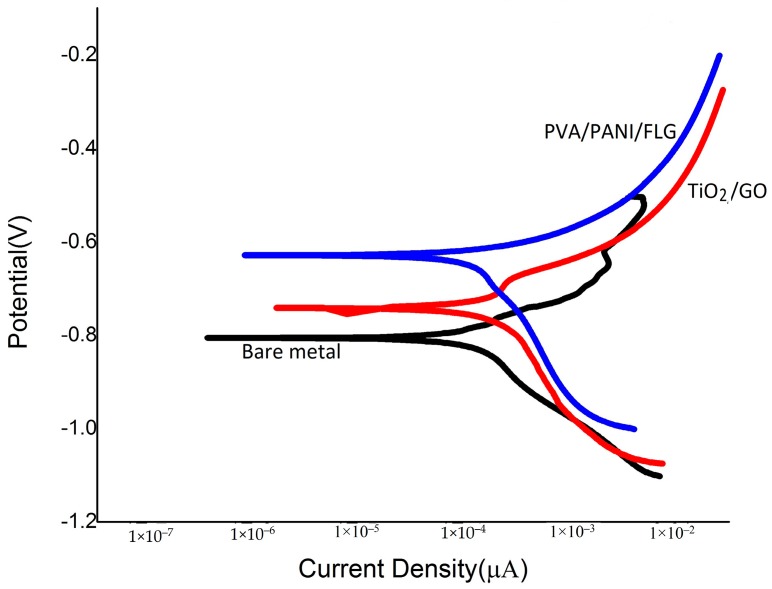
Tafel Scans in “produced water”: Current density in µA/cm^2^; Potential (V vs. SCE).

“E-log i” plots of bare metal and the coated surfaces exposed to “produced water” are shown in Figure 8, and the corrosion rates data are summarized in Table 5. The data reveal that the polymeric coating offered a significant decrease in the corrosion rate of about 1/7th of the rate without coating, whereas the ceramic base coating reduced the corrosion rate to about one half. The Tafel plots also support the previously mentioned indicators of the coatings’ performances in “produced water”.

**Figure 9:**
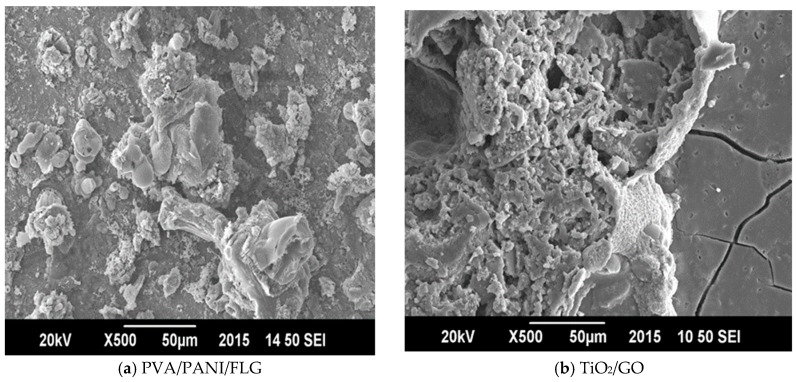
SEM micrographs of coated samples exposed to “produced water”. (**a**) PVA/PANI/FLG; (**b**) TiO_2_/GO.

Corrosion rates of bare metal and the coated surface exposed to seawater and the “produced water”, mentioned in Table 3 and Table 5, respectively, reveal that PVA/PANI/FLG coating sustained protection to a better extent in “produced water” compared with seawater, whereas the ceramic TiO_2_/GO coating behaved the other way round, presumably due to higher capacitance and pore resistance of TiO_2_/GO coating. SEM micrographs of the coated samples are shown in Figure 9, which reveals deformation and rupturing of the coating after exposure to the produced water. This degradation explains that the pores in the coating widens up, thereby leading towards corrosion of the substrate. 

## 4. Conclusions

It was concluded that both polymeric-based as well as ceramic-based coatings provided protection against environmental attacks posed by seawater and “produced water”, however, the degree of resistance varied between the two electrolytes. In the seawater environment, the PVA/PANI/FLG coating reduced the corrosion rate to about 52% compared with bare metal whereas in “produced water” the decrease in corrosion rate was up to 86%. The TiO_2_/GO coated surface offered a reduction in corrosion rate in seawater to about 94% whereas in “produced water”, the decrease in corrosion rate was about 57% compared with the bare metal. The porosity and coating capacitance plays a vital role in obtaining the abovementioned values, since a greater number of pores means a greater number of ions that diffuse through the coating, which exposes more surface area of the metal to corrosion reaction. Therefore, due to this mechanism, the coating which shows better pore resistance in any particular environment performed better compared with the one with the lesser value of coating resistance.

## Figures and Tables

**Table 1:** Materials used for PVA/PANI/FLG coating.

Material	Manufacturer
Polyvinyl alcohol	ERKOL
Polyaniline	Prepared in lab
Few layer graphene	I. Janowska

**Table 2:** Pore resistance and coating capacitances in seawater.

PVA/PANI/FLG		TiO_2_/GO	
R_pore_	4.219 ohms	R_pore_	7.626 ohms
C_c_	24.87 × 10^−3^ F	C_c_	11.50 × 10^−3^ F

**Table 3:** Corrosion rates in seawater (mpy).

Bare Metal	PVA/PANI/FLG	TiO_2_/GO
19.56	9.477	1.315

**Table 4:** Pore resistance and coating capacitances in Produced water.

PVA/PANI/FLG		TiO_2_/GO	
R_pore_	303.9 × 10^−3^ ohms	R_pore_	166.1 × 10^−3^ ohms
C_c_	17.59 × 10^−3^ F	C_c_	28.79 × 10^−3^ F

**Table 5:** Corrosion rates in “produced water” (mpy).

Bare Metal	PVA/PANI/FLG	TiO_2_/GO
21.76	3.140	9.315
